# Functional assessments for decision-making regarding return to sports following ACL reconstruction. Part II: clinical application of a new test battery

**DOI:** 10.1007/s00167-015-3546-3

**Published:** 2015-02-28

**Authors:** E. Herbst, C. Hoser, C. Hildebrandt, C. Raschner, C. Hepperger, H. Pointner, C. Fink

**Affiliations:** 1 Department of Trauma Surgery and Sports Medicine, Medical University Innsbruck (MUI), Anichstraße 35, 6020 Innsbruck, Austria; 2 Department of Orthopaedic Sports Medicine, Technical University Munich, Ismaninger Str. 22, 81675 Munich, Germany; 3 Sportsclinic Austria, Olympiastraße 39, 6020 Innsbruck, Austria; 4 Department of Sport Science, University of Innsbruck, Fürtstenweg 185, 6020 Innsbruck, Austria; 5 OSM Research Foundation, Olympiastraße 39, 6020 Innsbruck, Austria; 6 Sports Physiotherapy Mag. R. Huber, Steinbockallee 31, 6063 Neu-Rum, Austria; 7 Research Unit for Orthopedic Sports Medicine and Injury Prevention, Institute for Sports Medicine, Alpine Medicine & Health Tourism/UMIT, Hall, Austria

**Keywords:** Back to sports, ACL, Test battery, Limb symmetry index, Hop test, Stability test

## Abstract

**Purpose:**

The purpose of this study was to utilize a novel functional test system to facilitate determining the time of return to sports following ACL reconstruction.

**Methods:**

Sixty-nine patients with unilateral ACL reconstruction were included in this pilot study. All the patients performed a standardized test battery consisting of one- and two-legged stability tests, counter movement jumps, speedy jumps, plyometric jumps and a quick feed test. The first test was administered on average 170.7 ± 75.1 days post-operatively, and the retest was administered on average 239.1 ± 79.7 days post-operatively. The values of the subtests were compared with the normative data of healthy gender- and age-matched controls to determine the functional capacities of patients following ACL reconstruction.

**Results:**

After the first and second test, 15.9 and 17.4 % of the patients met the criteria for a “return to non-competitive sports”. One patient fulfilled the criteria for a “return to competitive sports” after the second test battery. The most limiting factor was a poor LSI value of <90 % if the dominant leg was involved and <80 % if the non-dominant leg was involved.

**Conclusion:**

This test battery demonstrates that, in terms of neuromuscular abilities, most patients, compared to healthy controls, are most likely not ready for a safe return to sports, even 8 months post-operatively. This should be considered in the future to determine when it is safe to return to sports and should avoid a premature return to competitive sports.

**Level of evidence:**

III.

## Introduction

During the last decade, anterior cruciate ligament (ACL) research has focused predominantly on anatomy and anatomic ACL reconstruction. Although the surgical procedure has been investigated thoroughly, there are unresolved problems. One major problem is the high ACL re-rupture rate. Webster et al. [31] recently reported an overall ACL re-rupture rate of 4.5 %. In young and active subjects, re-rupture rates of up to 20 % have been reported. One factor that might contribute to such high ACL re-rupture rates is a premature return to sports activities. According to Webster et al. [31], 50 % of ACL graft ruptures occur during the first year after primary ACL reconstruction. There is general agreement that professional and high-level recreational athletes must undergo ACL reconstruction to return to the pre-injury level [21]. Currently, at least 6 months are recommended before patients are allowed to return to contact or pivoting sports; although there is little firm evidence regarding the safe return to play [15], many athletes are pressured to make their comeback as soon as possible following surgery.

There are several relevant factors for a safe return to sports; however, some factors are more practicable than others. One important factor concerns the strength and maturation of the ACL graft. The graft undertakes a remodelling process during which the mechanical properties are affected. Most of the knowledge regarding the remodelling process is based on animal studies [8, 18, 27], and the results from animal models are not directly applicable to humans. From human biopsy studies, it is known that the remodelling process is similar in animal models and humans; however, the timeline is variable and unpredictable [14, 33]. Currently, clinical and/or functional predictors are relied upon to determine a safe return to sports. One important predictor to determine the safe return to sports after ACL reconstruction is the patient’s functional capacity. Even professional athletes with access to intensive rehabilitation and training programs have functional, neuromuscular and postural deficits following surgery, possibly leading to a higher ACL re-rupture risk. Several test protocols have been designed to provide objective measures, which should facilitate deciding when a return to contact or high-risk pivoting sports is relatively safe. Test protocols typically consist of laxity measurements and subjective scores as well as various jumping and strength tests [4, 5, 11, 23, 25]. Most of these protocols require expensive equipment or are extremely time-consuming or excessively complex for implementation in daily clinical practice [22]. Nevertheless, probably more than 90 % of the patients return to sports without any objective functional evaluation after ACL surgery. This might be a reason of such high ACL graft rupture rates. Therefore, a novel standardized test battery that is simple to use and does not require excessive equipment or a large amount of time or space was developed [34].

In this pilot study, the test protocol was used for the first time to evaluate the functional abilities of a group of patients following ACL reconstruction. The test battery covers different neuromuscular and coordinative skills and allows comparison to normative data of healthy subjects. It can be used in a routine fashion to objectively determine the earliest time point when patients are ready to safely return to sports following ACL reconstruction.

## Materials and methods

Sixty-nine patients, 27 (39.1 %) female (mean age 20.9 ± 7.8 years) and 42 (60.9 %) male (mean age 21.5 ± 5.7 years), were included in this prospective study. Thirty (43.5 %) patients had injured their non-dominant leg, whereas 39 (56.5 %) had injured their dominant leg. All the patients underwent a standardized early rehabilitation protocol. The time point for the first test was determined by the patient (“when he or she felt ready”), the treating surgeon and the physiotherapist as well as being by subjective and objective criteria (no swelling of the knee, full range of knee motion and the ability to safely perform all the functions required by the tests in physical therapy). The patients performed the test battery on average 170.7 ± 75.1 (range 100–494) days following unilateral ACL reconstruction and were retested on average 239.1 ± 79.7 (range 145–574) days post-operatively. The test and retest were used to determine whether a return to prior sports activity could be recommended.

**Fig. 1:**
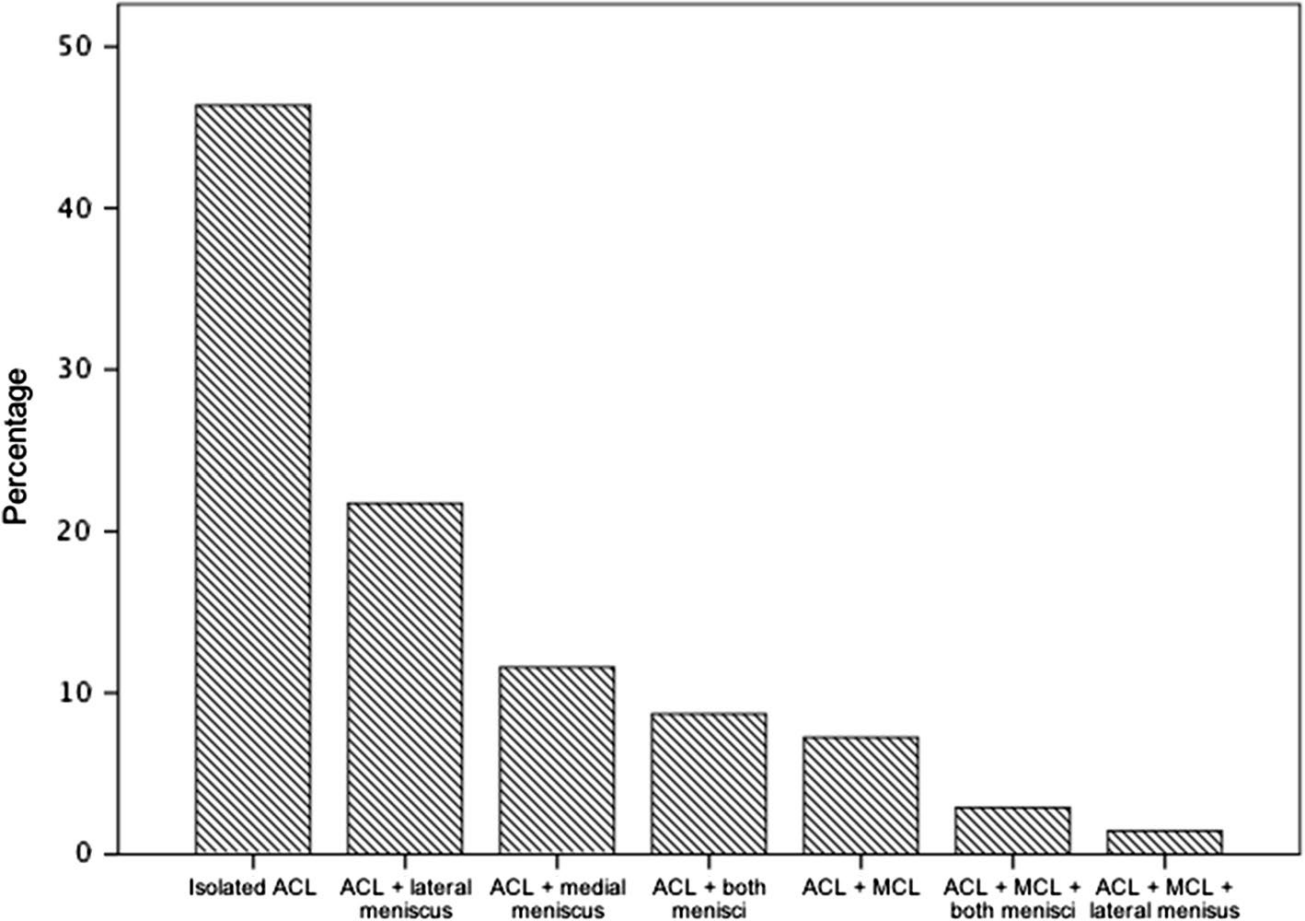
Injury pattern distribution within the study cohort shown as percentage

The injury patterns of the study cohort are presented in Fig. 1. For the ACL reconstructions, hamstring tendon grafts were used in 47 (68.1 %) cases, quadriceps tendon grafts in 12 (17.4 %) and bone patellar tendon bone grafts in 10 (14.5 %) patients. In 12 (17.4 %) of the 69 patients, the surgery was an ACL revision.

The following inclusion criteria were used: (1) unilateral ACL reconstruction with or without combined meniscus repair or partial meniscus resection as well as combined conservatively treated MCL injuries, (2) between 10 and 50 years of age at surgery, (3) a completed test and retest and (4) expected high patient compliance. The following exclusion criteria were used: (1) bilateral ACL tears, (2) multi-ligament reconstructions, (3) concomitant MCL reconstruction or repair, (4) clinically relevant cardiovascular history and (5) clinically relevant neurological and neuromuscular disorders.

### Description of the test battery

The test battery was previously described in detail [34]. The test battery “back in action” can be accomplished in 45 min and only needs little equipment and one room. It consisted of the following subtests: a two-legged (TL-ST) and one-legged stability test (OL-ST), a two-legged (TL-CMJ) and one-legged counter movement jump with height and power calculations (OL-CMJ), speedy jumps (OL-SY), plyometric jumps (TL-PJ) and a quick feed test (TL-QFT) [34].

The test–retest analysis of this new ACL test battery resulted in an interclass correlation coefficient between 0.688 and 0.921 for all the subtests [34].

The values of all the tests were categorized into five groups from “very good”, “good”, “normal”, “weak” and “very weak” according to the age- and gender-matched normal data of 434 healthy subjects. The categorizations considered the gender, patient age and leg dominancy. For the calculation of the limb symmetry index (LSI) of the one-legged tests, the resulting absolute value of the injured leg was divided by the value of the non-affected leg and multiplied by 100. For the stability, quick feet and speedy tests, lower values were considered better than higher values, and the calculation of the LSI was different. For these tests, the LSI was calculated by dividing the measured value of the non-affected leg by the value of the injured side and multiplying by 100. The different LSI calculations were performed to achieve comparable and consistent values for all the single-legged tests. With our adaptation of the LSI formula for those tests, the LSI for the injured leg is always suspected of being inferior to the unaffected side.

### Objective criteria for a return to sport

For a recommendation for a safe return to sports, a patient was required to score at least “normal” on any of the subtests. The patients who intended to return to competitive high-risk sports (e.g. alpine skiing or soccer) were required to score values that were at least regarded as “good” within the normative values (Table 1). An LSI >90 % for the dominant leg and an LSI >80 % for the non-dominant determined the time for a return to play.

**Table 1:** Objective criteria patients had to fulfil for a “safe” return to play compared to normative values of healthy controls

Categories of norm data	Competitive athletes	Recreational athletes
Very good	X	X
Good	X	X
Norm		X
Weak		
Very weak		
LSI dominant leg (%)	≥90	≥90
LSI non-dominant leg (%)	≥80	≥80

The procedures were reviewed and approved by the Board of Ethical Questions in Science of the University of Innsbruck.

### Statistical analysis

For the statistical analysis, SPSS^®^ 20.0 (IBM SPSS Statistics, New York, USA) for Mac software was used. The normal distribution was tested and confirmed with the Kolmogorov–Smirnov test for the metrical data and with the Chi-squared test for the nominal data. The quantitative parameters were evaluated with the calculation of the mean and standard deviations (SDs). To determine possible differences between the test values of the patients who were ready for a return to play and the patients who had deficits, the Student’s *t* test or Mann–Whitney *U* test were used, depending on the data distribution. To evaluate the progress during physical therapy between the tests and retests, matched pair analyses using the Student’s *t* test or the Wilcoxon test, depending on the data distribution, were performed. All the measurements are expressed with ±1 SD. Statistical significance was accepted for *p* ≤ 0.05.

A post hoc power analysis using G*Power 3.1.9.2 (Franz Paul, Kiel, Germany) was used to determine the power of the present study. Based on the results of the Student’s *t* test and Mann–Whitney *U* test, an effect size of 0.35 was calculated. With the underlying effect size, an *α* of 0.05 and a study group of sixty-nine patients, a power of 0.82 was calculated.

## Results

The use of this novel test battery was found to be feasible for all the patients, and no injuries were encountered throughout the test performances. The required test time per patient was between 45 and 60 min, including the warm up period.

After completing the test battery for the first time (approximately 5.6 months post-surgery), only 11 (15.9 %) of the 69 patients fulfilled the criteria determining a safe return to sports, indicating that they scored at least “good” or “normal”. At this time, none of the 63 patients who wanted to participate in competitive sports fulfilled the required criteria.

**Fig. 2:**
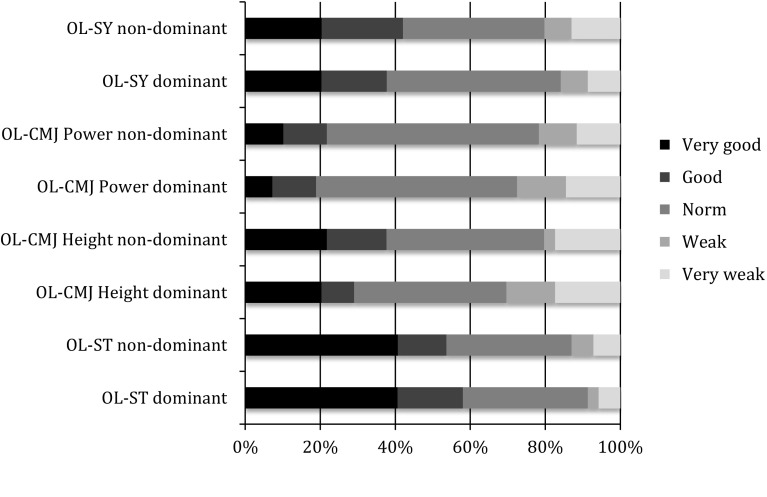
Single-legged subtests: performance reached at the initial test shown as percentage

**Fig. 3:**
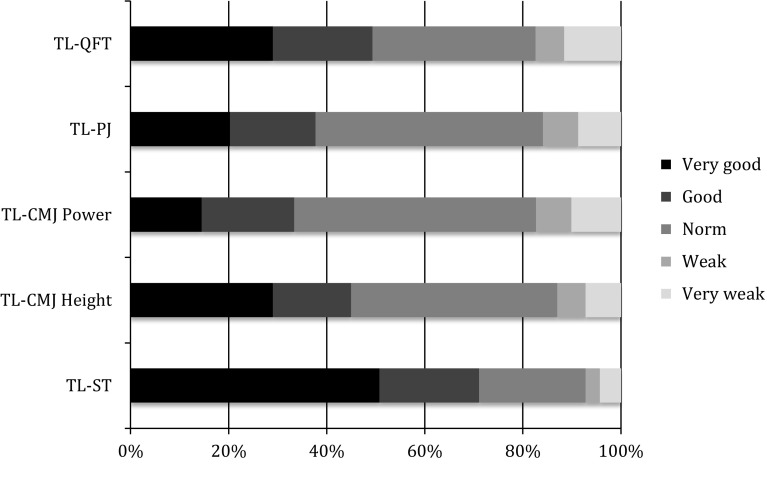
Single-legged subtests: performance reached at the initial test shown as percentage

The most limiting factor for a return to sports at this time point was the LSI. The pre-defined criteria for a safe return to play were LSI values >90 % if the dominant leg was affected and >80 % if the non-dominant leg was affected. For the OL-ST, OL-CMJ for height and power and the OL-SY, 17.4, 40.6, 39.1 and 5.8 % of the patients did not meet those criteria. Figures 2 and 3 show the details of each subtest compared to those of a group of healthy subjects.

After completing the test battery for the first time, two subtests showed significantly (*p* < 0.05) different results. The absolute values for the OL-ST of the dominant leg (*p* = 0.048) and the LSIs for the OL-CMJ for height and power (*p* = 0.013, *p* = 0.022) differed significantly between those patients who were ready and were not ready to return to play.

After completing the test battery for the second time (approximately 8 months following ACL reconstruction), 12 (17.4 %) patients were ready for a return to sports, and one patient met the criteria for a safe return to competitive sports.

The LSI was the most limiting factor at this time point for a return to play. For the OL-ST, OL-CMJ for height and power and the OL-SY, 13.0, 24.6, 37.7 and 1.4 % of the patients did not meet those criteria (Table 2).

**Fig. 4:**
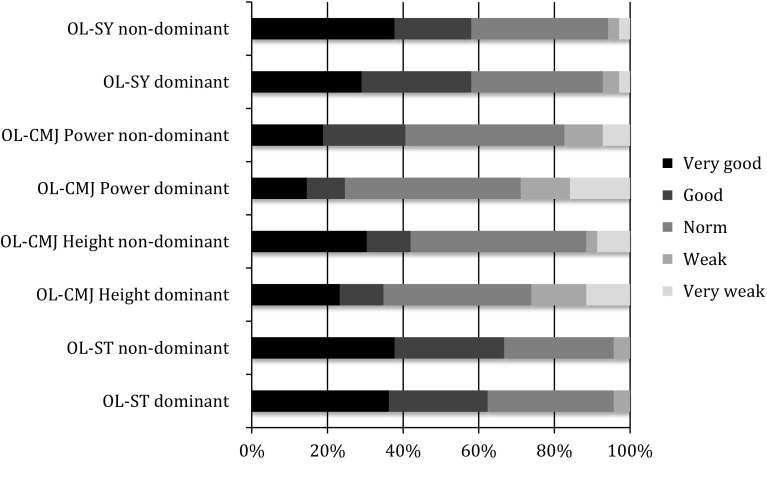
Single-legged subtests: performance reached at the retest shown as percentage

**Fig. 5:**
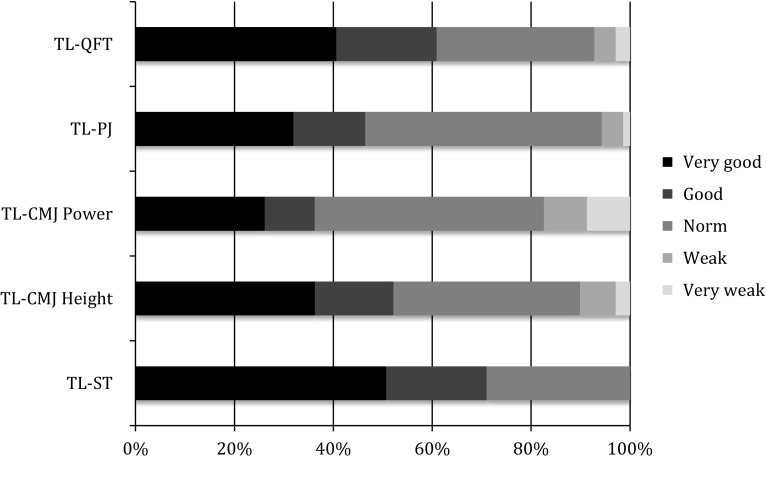
Two-legged subtests: performance reached at the retest shown as percentage

Figures 4 and 5 summarize the patients’ scores in the retest compared to those of the group of healthy subjects.

In the retest, significant differences between the patients who were ready and were not ready to return to play could be detected by the TL-CMJ for height and power (*p* = 0.025, *p* = 0.014), the OL-CMJ of the dominant leg for height (*p* = 0.003) and for the TL-PJ (*p* = 0.000). The subtest values showed no significant differences (n.s.) between the groups.

The evaluation of the test and retest performance of the study cohort showed a significant (*p* < 0.05) improvement over time in most subtests (Tables 3, 4).

**Table 2:** LSI for all one-legged subtests of test and retest shown as mean values ± SD

	Absolute values		*p* value
	Test	Retest	
LSI OL-ST	102.6 ± 15.3	99.9 ± 12.7	n.s.
LSI OL-CMJ height	87.4 ± 20.7	93.1 ± 15.4	0.016*
LSI OL-CMJ power	89.9 ± 22.7	93.3 ± 20.0	n.s.
LSI OL-SY	97.5 ± 13.9	98.7 ± 13.1	n.s.

*p* values marked with * indicate statistical significance

**Table 3:** LSI with respect to dominant and non-dominant side for all one-legged subtests of test and retest shown as mean values ± SD

	Absolute values		*p* value
	Test	Retest	
OL-ST dominant	2.1 ± 0.5	2.0 ± 0.4	n.s.
OL-ST non-dominant	2.1 ± 0.5	1.9 ± 0.3	0.004*
OL-CMJ height dominant	21.5 ± 4.5	22.5 ± 5.3	0.018*
OL-CMJ height non-dominant	20.9 ± 5.7	22.3 ± 4.3	0.010*
OL-CMJ power dominant	27.3 ± 5.9	27.9 ± 7.9	n.s.
OL-CMJ power non-dominant	26.6 ± 7.9	28.0 ± 6.4	n.s.
OL-SY dominant	5.9 ± 1.1	5.4 ± 1.1	0.000*
OL-SY non-dominant	5.9 ± 1.3	5.5 ± 0.9	0.003*

*p* values marked with * indicate statistical significance

## Discussion

The most important finding of the present study was that approximately 8 months following ACL reconstruction, 82.6 % of the patients had functional deficits in one or more parameters compared to a group of healthy age- and gender-matched subjects. Additionally, only 1.5 % of the patients were ready for a return to competitive sports, if strict criteria were applied.

The test battery “back in action” is practical since it can be accomplished in 45 min and only needs very little equipment and space. Furthermore, due to the software used, a quick evaluation and feedback of the patient’s performance is possible.

**Table 4:** LSI for all two-legged subtests of test and retest shown as mean values ± SD

	Absolute values		*p* value
	Test	Retest	
TL-ST	2.1 ± 0.5	1.9 ± 0.4	0.023*
TL-CMJ height	38.4 ± 5.7	40.1 ± 6.6	0.000*
TL-CMJ power	44.4 ± 7.9	45.7 ± 9.9	n.s.
TL-PJ	2.1 ± 0.5	2.2 ± 0.5	0.002*
TL-QFT	8.5 ± 1.7	8.0 ± 1.2	0.000*

*p* values marked with * indicate statistical significance

Webster et al. [31] recently published a post-ACL reconstruction graft rupture rate of 4.5 % during a 3-year follow-up. Patients under 20 years of age have a particularly high incidence of ACL re-rupture. After 5 or more years, up to 11 % of patients sustained a re-rupture and up to 16 % had a contralateral ACL injury [6, 32]. Although those studies document the re-rupture rate over a 3- to 5-year period, many ACL re-ruptures occur within the first year after surgery [26, 31]. Several studies found a higher risk of ACL re-rupture if patients participated in a high-risk sport that includes pivoting or cutting movements [16, 26, 31].

Although up to 90 % of the patients return to play after an ACL surgery [2, 10, 12, 19], only 50 % return to their pre-injury activity level [2, 3, 10, 19]. These studies demonstrate that although many patients return to play after ACL reconstruction, the timing might be premature with respect to their functional abilities or the mechanical properties of the graft. These factors might place patients at a significant risk of re-injury. Most of the knowledge regarding the remodelling process originates in animal studies. Those studies show that the biomechanical properties of a graft are at their weakest between 6 and 8 weeks post-operatively, followed by a slow increase in strength. The graft could resist physiological loads 6–8 months post-operatively [8, 18, 27]. Physiological remodelling in humans might be slower [14, 33]. Restoration of functional capabilities following ACL reconstruction is crucial because the mechanical characteristics of a healing graft could not be safely characterized.

The time point for a safe return to competitive sports is highly controversial. Currently, a minimum of 6 post-operative months is recommended before returning to pivoting and competitive sports [12, 15].

Despite intensive physical therapy following ACL surgery, functional deficits might be present much longer than surgeons or patients have previously thought. We demonstrated in our study that only 17.4 % of the patients did not show functional deficits 8 months post-surgery. The remaining 82.6 % of the patients had a functional deficit in at least one subtest, and a safe return to sports could not be recommended.

Laboute et al. [16] found that patients who returned to competitive sports within 7 months post-operatively had a significantly higher risk of ACL re-rupture than did the patients who returned to play later (*p* = 0.014). This finding is supported by the fact that patients who return to strenuous activities following ACL reconstruction have a higher risk of ACL re-rupture and contralateral ACL injury [26]. The experience of our study as well as that of other studies questions the value of a time-based return-to-sports recommendations and favours the use of a criteria-based approach [16, 26].

This high incidence of graft failure and contralateral ACL rupture during the first year after ACL reconstruction might be related to insufficient graft strength as well as to limb asymmetries or neuromuscular deficits. Ageberg et al. and Augustsson et al. [1, 4] reported that the LSI value could detect functional deficits. In our study, the most limiting factor for a return to play was an LSI below 90 % for the dominant leg and 80 % for the non-dominant leg on the test and retest after 5.6 and 8 months, respectively. Poor LSI values for the jumping, stability and quick feet tests are supported in the literature. In our study, the mean LSI value of the CMJ was approximately 93 %. Thomeé et al. [29] found post-operative LSI values for the CMJ of 77.3 and 88.4 % at 6 and 12 months, respectively. In their study, an LSI value of ≥90 % was not achieved until 24 months post-operatively. This finding is in accordance with the data published by Myer et al. [20]. At 10 months post-surgery, they found an LSI of 89 % on the vertical jump tests. Additionally, the LSI values for the hop tests correlated with the self-reported outcome measures, as well as with the return to the pre-injury level after ACL reconstruction [3, 17].

In this test battery, over 90 % of the patients achieved values within the “norm” compared to the healthy controls on the stability tests. Webster et al. [30] demonstrated that subjects with an ACL-reconstructed knee have inferior stabilizing abilities compared to the abilities of healthy controls even 2.5 years after surgery. Their findings are supported by other researchers [7, 24]. A systematic review of Howells et al. [13] found high discrepancies for the stability and postural performance of patients following ACL reconstruction. They documented that ACL surgery leads to limitations in the single-legged and two-legged stability tests. This finding is in good accordance with our data.

Most studies investigating the neuromuscular outcome following ACL surgery use various hop tests [9]. Myklebust et al. [21] found that even professional athletes with their extensive rehabilitation and training programs have deficits in the hop test performance in the reconstructed limb compared to the performance on the uninjured side. Further, the hop test performance correlates with the ability for a return to play [28].

A test battery for the determination of a “safe” return to play must assess postural abilities and hop tests as well as speed and muscle strength testing.

This study has several limitations. This pilot study included only a limited number of subjects, and no standardized clinical data in terms of knee scores or instrumented ACL laxity tests were gathered. To show a positive influence regarding re-rupture, a higher number of patients and longer follow-up are needed.

To the best of our knowledge, this study is the first to use a test battery to compare normative data of age- and gender-matched healthy controls to that of subjects who had undergone ACL reconstruction. This comparison allows a categorization for every subtest to determine whether patients continue to have functional deficits or whether they have overcome their functional deficits. Based on the results of an initial test at 4–6 months, recommendations regarding future rehabilitation and training might be offered. The patient as well as the physician could observe improvement during the rehabilitation process. This pilot study shows that in many patients, their perception on their functional abilities diverges markedly from their test results. For the patient, an objective test result provided motivation for further training and was crucial for an awareness of the risks associated with a premature return to sports activity.

Based on our results, surgeons have to be more restrictive in determining a return to sports. Answering the crucial question of when to safely return to play is necessary and can be made possible using a standardized test battery with objective comparison with healthy controls. The future will show whether such test batteries are helpful to prevent re-injuries after ACL surgery due to a too premature return to sports.

## Conclusion

Approximately 8 months following ACL surgery, a comparison of the results on our novel test battery to the normative data of healthy controls indicated that only 17.4 % of the patients were ready for a return to play. Of the 69 patients, only one patient met the criteria for a return to sports on a competitive level despite participation in a controlled rehabilitation program. The test battery was shown to be safe and extremely helpful in counselling a patient with respect to further training and the timing of a return to sports.
